# Exploring values among three cultures from a global bioethics perspective

**DOI:** 10.1080/11287462.2021.1879462

**Published:** 2021-02-01

**Authors:** Nico Nortjé, Kristen Jones-Bonofiglio, Claudia R. Sotomayor

**Affiliations:** a University of Texas MD Anderson Cancer Center, Houston, Texas, USA; b The University of the Western Cape, Bellville, South Africa; c Lakehead University Centre for Health Care Ethics, Thunder Bay, Canada; d Associated Medical Services (AMS Healthcare) Phoenix Fellow, Toronto, Canada; e Georgetown University, Washington, DC, USA; f Research Scholar of UNESCO Chair in Bioethics and Human Rights, Rome, Italy

**Keywords:** Culture, global bioethics, Ojibway, Mayan, Xhosa

## Abstract

The United Nations Educational, Scientific and Cultural Organisation’s (UNESCO) Declaration on Bioethics and Human Rights refers to the importance of cultural diversity and pluralism in ethical discourse and care of humanity. The aim of this meta-narrative review is to identify indigenous ethical values pertaining to the Ojibway (Canada), Xhosa (South Africa), and Mayan (Mexico and Central American) cultures from peer-reviewed sources and cultural review, and to ascertain if there are shared commonalities. Three main themes were identified, namely illness, healing, and health care choices. Illness was described with a more complex and dynamic picture than from the western view, as illness is not considered to be one dimensional. Healing needs to take place on various levels in order to restore a state of equilibrium between the different spheres. Health care choices were also considered from a multi-level perspective. In all three of the indigenous cultures explored, good decision-making is seen to have occurred when choices are informed by commitments to one’s moral and ethical responsibilities towards the community, nature, and the spirit world.

## 1. Introduction

With globalization, the rise of pandemics, and natural disasters, the deep connections between individuals, communities, and their traditions and cultures, can no longer be overlooked. UNESCO’s (2005) Declaration on Bioethics and Human Rights succinctly refers to the importance of cultural diversity and pluralism in ethical discourse and care of humanity at large. The Declaration is based on the tenets of global bioethics, which is a response to increased awareness of the interrelatedness of individuals and their ethical dilemmas, perceived cultural differences, and unique contributions from people around the globe (ten Have & Gordijn, 2013). Informed practitioners of clinical ethics will appreciate that the biased use of secular values and principles (such as individualism, autonomy, etc.) in healthcare settings often leads to the discouragement and even abandonment of ethical values specific to indigenous cultures, which raises health justice concerns. According to ten Have and Gordijn (2013, p. 635) “the confrontation with different ethical traditions and cultures is challenging ethics to rethink and transform its content, character, methods and sources of validation”.

For example, some scholars have observed that in many indigenous cultures, morality and ethical thinking is shaped by the specific conditions under which people live, affected by the community, the seasonal variations, and is even expanded to include animals and elements of the natural environment (Barrera-Bassols & Toledo, 2005; Lovin & Reynolds, 1992). Indigenous peoples often have a strong commitment to community relationships and involvement, which challenges the presumptions of the western bioethical paradigm that privileges the individual autonomous subject, prescriptive procedures, and discursive norms (Stevenson, 2016). The influences, as well as points of convergence among western and eastern religious views, on bioethics have been widely studied by various scholars (Tham, 2014; Vries, 2015), however little is known about the contribution of indigenous cultures to the global bioethics discourse (Tham, 2014; Vries, 2015). This identified gap in global bioethics, as well as increased interest in other academic fields (such as anthropology; psychology; sociology; and, philosophy) to explore and acknowledge the ancient wisdom of indigenous knowledge, has created an awareness that the global ethics movement may benefit from a more holistic perspective on ethics and a broader understanding of decision-making by including indigenous frames of reference.

The aforementioned need is exacerbated in times of crisis (such as the COVID-19 pandemic) when members of indigenous cultures, often times, have a different outlook on the place and the power of the individual in decision-making (Weaver, 2012). Literature indicates (Bird, 2002; Castor et al., 2006; Swan et al., 2006; Weaver, 2012) that during times of traditional decision making, more goes into the process than merely considering what is medically in the best interest of the singular patient/inflicted one. This type of approach is challenged when indigenous people, especially those who live in rural areas, need to seek health care in urban settings, where the *modus operandi* is geared toward individualism (Weaver, 2012). The aim of this meta-narrative review is to identify ethical values pertaining to decision-making among the Ojibway (Canada), Xhosa (South Africa), and Mayan (Mexico and Central American) cultures from peer-reviewed sources, and to ascertain if there are shared commonalities between the three indigenous cultures, and then to extrapolate this into a global bioethics approach.

We acknowledge that it may be a limitation to focus on only three cultures. However, given our individual working experiences with these three cultures respectively, and through the trust of elders expressed during informal conversations, it was decided to focus the literature research on these three cultures. It is not the intent of this paper to illustrate that one value system is superior to, or in contrast of, the other. However, the aim is to illustrate the importance of shared decision-making between different worldviews and how these differences do not need to lead to conflict, but rather may build capacity and perhaps even synergy. We recognize the limitations of the use of academic findings from the recent past that may have had other influences (e.g. political, religious, etc.) on the understanding of contemporary indigenous cultures and communities. Upon discussion, some “ideals” will be highlighted that may have some distance from the practical realities of life as experienced by indigenous people (e.g. poverty, racism, discrimination). Analysing the results of the literature review and receiving feedback from members of these cultures, the authors hope to contribute unique positions to the current discourse on global bioethics, focusing on decision-making.

## 2. Methodology

### 2.1. Method

Meta-narrative as a methodology tasks itself to synthesize information gained from diverse studies by virtue of extensive literature reviews (Greenhalgh et al., 2005; Wong et al., 2013). A general scoping exercise is conducted through which the researcher identifies relevant, albeit divergent, fields of study which could inform the research question at hand. Upon identification of the fields (e.g. anthropology, music, history, arts, medicine, language, philosophy, etc.) an extensive search is conducted for relevant articles which would fit specific parameters. This strategy is highly suited to comparing and contrasting information from different fields of expertise and to distil new information (Greenhalgh et al., 2005; Wong et al., 2013). Once all the articles were read by the authors, all relevant information was coded and three main themes were identified. As a supplementary quality check the authors also involved indigenous scholars from each culture who evaluated the results and commented on accuracy and applicability.

### 2.2. Data sources and searches

A comprehensive search in English was done using the EBSCOHost platform, indexed after 1 January 1967. The databases included in the search included: Academic Search Complete, Africa-Wide Information, AHFS Consumer Medication Information, American Doctoral Dissertations, Art & Architecture Source, Art Index Retrospective, ATLA Religion Database with ATLASerials, Communication & Mass Media Complete, GreenFILE, Health Source – Consumer Edition, Health Source: Nursing/Academic Edition, Humanities Source, MasterFILE Premier, MEDLINE with Full Text, PsycARTICLES, PsycINFO, RILM Abstracts of Music Literature (1967 to present only), and SocINDEX with Full Text. The search combined subject headings in seven inclusion categories (i.e. values, personhood, leadership, cultural norms, beliefs, social issues, culture, and knowledge), for each of the cultures under study (i.e. Mayan; Ojibway; and, Xhosa). Furthermore, narrative studies in English and Spanish, indexed after 1 January 1967, pertaining to the fields of Cultural Anthropology; Medical Anthropology; Religion; History; Literature (folklore); Sociology; Philosophy; and, Indigenous Knowledge were also included.

### 2.3. Study selection

The systematic search identified a total of 553 articles (Mayan = 163; Ojibway = 189; and Xhosa = 201). A grading matrix was used where all authors rated each one of the abstracts from the 553 articles and identified them as probably eligible, possibly eligible, or ineligible. The inclusion criteria consisted of satisfying the majority of the authors (at least 2 of the 3) and that the abstract included enough information about one or more of the aforementioned inclusion categories (i.e. values, personhood, leadership, cultural norms, beliefs, social issues, culture, and knowledge). A total of 371 articles were deemed by the authors to be ineligible (Mayan = 107; Ojibway = 126; and Xhosa = 138) as they did not pertain to any of the categories. A total of 182 articles (Mayan = 56; Ojibway = 63; and Xhosa = 63) were found to be eligible and were downloaded. These articles were read by the authors and a final count of 52 were found to have information that was significant to the research question at hand namely ethical values related to decision making (Mayan = 15; Ojibway = 16; and Xhosa = 21). The main reasons for further exclusion of articles were: a focus on agriculture and a Mayan population from Chiapas, which were infiltrated by guerrilla warriors and lost many of their traditional ways of life; a focus on circumcision and also agricultural traditions among the Xhosa; and finally, a focus on teaching/education/curriculum, natural resource management/agriculture, or very historical/anthropological perspectives (including treaties) among the Ojibway people. A general evaluation of the reference lists of the 52 articles identified only one additionally eligible article for the Mayan group, which was included in the final analysis.

The experts who were asked to evaluate the results, discussion, and conclusion of this paper, were native tothe specific culture and had academic and experiential backgrounds to critically evaluate the paper.

## 3. Results

From a global bioethics perspective, it is the intention for this paper to bring forward a sample of three indigenous cultural perspectives to expand the dialogue on quality health standards and ethical decision making. The research question focused on whether there are shared commonalities among the three indigenous cultures’ approaches to decision-making. Three main themes were identified from the data sources (based on repetitive occurrence in all the articles), namely illness, healing, and healthcare choices. Following is a summary of the findings according to the three main themes, as presented in each culture.

### 3.1. Illness

#### 3.1.1. Mayan

Mayan life is interrelated, with the physical world as well as the gods from the sky, earth, and underworld. This interconnectedness is reflected in the Mayan view of illness, where some illnesses are considered natural or “earthly” illnesses, while others are considered to be provoked by supernatural forces, often referred to as bad winds (Barrera-Bassols & Toledo, 2005).

Earthly illnesses are often connected to the humoral system, where the illness is either classified as being humoral “hot” or “cold.” For example, diarrhoea is considered to be a “cold” illness, while dysentery (bloody diarrhoea) is classified as “hot.” The Mayans hold that when a person is in a “hot” state, consuming something “cold” may lead to illness (Ankli et al., 1999; Christenson, 2007). The Mayan tradition furthermore holds that bad winds, which were created in ancient times when a sorcerer became angered at the inhabitants of a village and decided to destroy them, can enter into the body of a person and make him ill (Ankli et al., 1999; Steele, 1977). Tradition also indicates that illness can be brought about by certain magic persons, who are born with the power to cause illnesses, simply looking at someone (Christenson, 2007; Steele, 1977). Since the Mayan culture believes that all things past and present are connected, misdeeds from the past can also create illnesses (Christenson, 2007; Steele, 1977).

#### 3.1.2. Ojibwa

As first described by Hallowell (1963), the traditional Ojibway perspective of illness has a personalistic theory of causation with both spiritual and moral components (Bishop, 2008; Garro, 1990). Since personhood in an Ojibway context includes more than just humans, illness can be caused by not only inappropriate human actions (*onjine*) but also by witchcraft (bad medicine) (Barkwell, 2005; Garro, 1990). It is interesting to note that males may experience illness due to direct or indirect contact with a menstruating woman (Garro, 1990; Johnson, 1997) as menstrual blood is regarded as a means of purification of toxins (Gaudet & Caron-Bourbonnais, 2015; Wilson, 2005). This type of exposure may also negatively impact a man’s success on a hunt or trap line, where contamination will lead to failure. Further explanations of illness could be due to influence(s) from outside the culture (e.g. “White Man’s” illnesses such as infectious diseases) or from no particular cause (*inaapine*) (Garro, 1990).

#### 3.1.3. Xhosa

Should a member of the Xhosa clan become ill, there is likely one of three reasons for the illness (Cumes, 1995; Jonas, 1985; Mbiti, 1989; Mlisa & Nel, 2013; Postel, 2010; Rice, 2015). The first reason is punishment by the ancestral spirits because a member, or the clan itself, neglected to adhere to the customs of the clan. The second reason is the work of witches or sorcerers who intend evil and misfortune to befall a member or the clan. The last reason is due to pollution which often takes on the form of bad blood (*umlaza *= menstruating). All three, but especially for the first two, indicate very powerful social controls and necessitates some kind of intervention by the individual and the clan.

### 3.2. Healing

#### 3.2.1. Mayan

Mayan beliefs hold that land and water are the first medicines that the gods of the sky and earth provide to counteract illness (Barrera-Bassols & Toledo, 2005; Christenson, 2007). The land provides herbal medicines and special diets to cure illnesses (Faust, 1988). The offering of sacrifices forms an important aspect of healing as it is seen as an exchange or gift to the “Spirit of the Land”, to re-establish the health of a sick person (Barrera-Bassols & Toledo, 2005; Christenson, 2007; Nash, 1967). It is commonly thought that blood (red) will nurture land, prevent diseases and/or help individuals to recuperate from disease. Together with these elements, depending on the severity of the illness, the sick will not be cured without the help of the lords of the winds (Christenson, 2007).

Both pre-colonization views on heating and cooling influences, and notions introduced by the colonizing Spanish pertaining to Hippocratic traditions, are combined in modern Maya medicine. The Mayan culture does not discriminate against western medicines (Anderson et al., 2004; Christenson, 2007), but it is still believed that the curing rituals need to be in coexistence with western medicine to have a complete effect. To illustrate this Faust (1988) argues that a local traditional healer is often said to be better able to cure chronic illnesses (e.g. attacks of nerves, rashes, psychological withdrawal, or disorientation), whereas the clinic doctor is best at injuries, suturing, and bone fractures, addressing contagious epidemics, fevers and vomiting, and for emergencies related to abnormalities of pregnancy and childbirth (Faust, 1988).

Since healing is a process of involvement from various role players, there are different types of medical providers. The H-men, who are not only healers but also specialists in religious rites, perform ceremonies addressing the rain-god to ask for protection of the *milpa* or the community (Christenson, 2007). The H'men or shaman (the middle man between spiritual forces and human beings) represents land-men connectedness to the spiritual realm (Barrera-Bassols & Toledo, 2005). The H-men’s deep knowledge of the family’s history and the interrelationship is an important element of his curing practice that involves a symbolic sensory experience including touch, taste, smell, hearing and sight, with the usage of herbs and faith (Faust, 1988). There are also the *hierbateros*, who specialize in herbal remedies; the *huesero* or *sobadores*, who give massages; and, the *parteras* or midwives (Christenson, 2007; Faust, 1988; Nash, 1967). All these members of the care team work together to heal an individual from illness.

#### 3.2.2. Ojibwa

Healing in traditional Ojibway contexts promotes balance and well-being on physical, mental, emotional, and spiritual levels (Bishop, 2008; Garro, 1990; Garro, 1996; Manitowabi & Shawande, 2011). The belief is held that in order to attain or regain health and restore balance the individual needs to focus on right living, spiritual intervention through ceremony, and natural medicines (Manitowabi & Shawande, 2011). Traditional practices for healing and well-being include healing ceremonies (*Midewiwin)* (Garro, 1990*)*.

Traditional healers may connect to an Ojibway person’s pain and illness through intuition and empathy (Barkwell, 2005; Bishop, 2008; Garro, 1990), without necessarily having to talk about the issues at hand. Two types of Ojibway healers are identified, namely those who heal as a gift (through visions/dreams) and those who learn to heal with traditional medicines (Garro, 1990). Additional to the aforementioned, healing can also be obtained from the blessings of other-than-human-persons (OTHPs) and therefore a connection to the spirit world through ceremony is often needed (Barkwell, 2005; Bishop, 2008; Garro, 1990; Hallowell, 1963).

#### 3.2.3. Xhosa

In the traditional Xhosa culture the first intervention to be sought if a member of the clan becomes ill is the services of an *igqira* (healer) (Cumes, 1995; Mlisa & Nel, 2013; Rice, 2015). The *igqira*’s role is seen on three levels, namely a priest, medical practitioner, and psychologist (Dovey & Mjingwana, 1985; Rice, 2015). The *iqgira* is tasked to communicate with the ancestors (through channelling; throwing of bones; or interpreting dreams) to understand what has upset them or who is bewitching the member of the clan (Cumes, 1995; Jonas, 1985; Ogungbile, 2008). Healing therefore necessitates a continued ritual process of correcting the disharmony generated by the spiritual, natural, mental, and social factors by appeasing the ancestors through sacrifices and divination (Adogame, 2008; Cumes, 1995).

A member of the clan does not have license to disagree with the advice of the *igqira* and would not contemplate to look for a second opinion, as the *igqira* has been divinely instructed and the ancestors have spoken (Postel, 2010). This illustrates the beliefs that may underpin a lack of willingness to disagree that is often seen in traditional Xhosa people when in relationship with medical practitioners. The values of obedience and respect are embodied, again, in the choices a member of the clan will make.

### 3.3. Healthcare choices

#### 3.3.1. Mayan

Healthcare choices are a complex endeavor for the Mayans because no decision is taken autonomously by one individual, but rather as a communal decision where the extended family and the H-men participate (Faust, 1988). Mental reasoning is not taken into consideration when making healthcare choices, because it is believed that the human heart is the receptor of the divine essence that comes from the Heart of the Sky and the Heart of the Earth, therefore it is only the heart that enables people to use their good sense and not the brain (Micalco, 2013).

Given the fact that the Mayan outlook on cosmology is informed by divine essence, it is their belief that they should not fight when it is time to die. No extra-ordinary measures have to be employed in the culture to save lives. At most, a person may, at any time, announce that their time has come to an end and retreat to their mat or hammock, where they would lie quietly, awaiting death. Immortality and an afterlife exist in the Mayan belief system (Steele, 1977).

#### 3.3.2. Ojibwa

Respect is a multi-facetted concept in the Ojibway culture which influences everyday actions, including healthcare choices. Socially approved displays of respect are part of a greater obligation to the spirit world for the gifts bestowed on Ojibway people (Garro, 1990). Respect can be shown by not interfering with the normal course of life, for example allowing babies and children to learn, play, and explore without reprimands (Hay, 1973), through gift giving (e.g. tobacco), keeping one’s home open to family and guests (Hadjiyanni & Helle, 2009), and honouring the four sacred directions (Johnson, 1997).

Because respect also extends to language and thoughts, a traditional approach to someone being diagnosed with an illness is to avoid the subject for the good of that person as well as the protection of others in the community (Barkwell, 2005). It is therefore not uncommon that healthcare choices are made with a sense of communion with all other relationships in mind, including the natural and the spiritual worlds (Bedard, 2008). Individuals who need to make choices will often consult elders for guidance, however an elder will never provide direct advice (Hay, 1973). After receiving a request for help, an elder may tell a story to the Ojibway person from which he/she must interpret the teaching with regards to their healthcare choices. This may be culturally misinterpreted as disengagement or apathy to take responsibility for healthcare choices, however respect and not forcing a given opinion extends to behaviour and attitudes as these can influence and shape reality in the Ojibway cosmological definition.

#### 3.3.3. Xhosa

In order to understand healthcare choices in the traditional Xhosa culture, it is paramount to understand that according to the Xhosa belief system, well-being, good fortune, and/or success are dependent on the individual, his/her family, as well as the clan’s satisfactory relationships with their specific ancestors (also referred to as the living dead or shades, hence the reason why a person’s shade is seen as a spiritual manifestation of the self) (Rice, 2016; Venter, 2013). These satisfactory relationships are deeply rooted in filial obedience and patriarchal authority, and are supported by a gerontocratic style of interaction between members of the clan and the elders (Rice, 2016). The values of honour and respect for elders would therefore often dictate members’ choices in life.

A significant influence on choices in life for a Xhosa person is the fear of bringing shame upon the clan (Rice, 2016). Shame is seen as that which is brought upon the clan in terms of illness or misfortune due to the violation of a taboo (Asante, 2008). A person who dishonours the ancestors or disrespect an elderly person (a taboo) can be the cause of punishment. This punishment can often result in collective condemnation. Since an individual only gets their sense of self-worth from acknowledgement of their standing in the clan, upsetting the equilibrium could be unsettling and can be a powerful force of social control in decision-making (Mlisa & Nel, 2013; Postel, 2010; Rice, 2016). It is because of this need for acceptance that individuals (especially men) in the Xhosa culture will withstand a substantial amount of physical and/or emotional suffering (even the loss of life at times) as proof that the individual has the ability to withstand difficult circumstances should they ever be tempted (Venter, 2013). This display of bravery is also pleasing to the ancestors. As Venter (2013) explains, suffering is often seen in the Xhosa culture as part of a higher purpose and members should welcome it in pursuit of acceptance by their group and to gain a better standing. Hence, suffering can assist in developing their personhood (Kubow & Min, 2016; Van Heerden, 2002).

Ultimately Xhosa beliefs are not only concerned about mortal life on earth in service of the clan (living and dead), but also about the transition to the world of the shades where they will be honoured by other clan members. The result, therefore, is that when an individual needs to make a decision pertaining to medical issues, he/she will defer it to the clan and call upon the elders to make the decision on his/her behalf, in order not to upset what is “good” for the clan as a whole. Honouring the clan by recognizing their role in the lives of individuals is greatly pleasing to the ancestors (Rice, 2016) and will therefore dictate the decision making of individuals.

## 4. Discussion

Global concerns about health and well-being demonstrate the interdependence of people in the world (ten Have, 2011), however traditional western approaches to bioethics and health decision making demonstrates a narrow mind-set that privileges autonomy and independent decision making. The World Health Organization (WHO) states that health inequities arise because of the circumstances in which people grow, live, work and age (2009), and efforts are being made to reduce those inequities and improve social development (e.g. the Millennium Development Goals [MDGs]) (Jacob, 2017). From a global bioethics perspective, globalizing the concerns of bioethics means that more attention must be paid to issues relevant to developing countries, and in particular to global inequities in health.

The findings in this study present a very different picture of health and illness; a complex, dynamic, and interdependent picture. Disease, in these three cultures, can be considered as more than just a state of physiological deterioration. Rather it can be seen as an imbalance between physical, environmental, social, mental, and/or spiritual spheres. This holistic view of disease is strongly related with their views on the interdependence of the human person with their community and the Cosmos that is ordained to promote, sustain and foster life. It is interesting to see that all three cultures share a perception that the disease and illness that befalls an individual always indicates that something is wrong on a larger scale, either in their human relationships or in their relationship with the Cosmos. This is in line with the World Health Organization's (WHO) definition of disease, which argues that health is “a state of complete physical, mental and social well-being, not merely the absence of disease or infirmity” (Scully, 2004, p. 651).

This definition given by the WHO attempts to give a holistic approach to what health is, however in practice, the biomedical explanation of disease still carries the most weight as can be seen in the International Statistical Classification of Diseases and Related Health Problems (ICD-10) used to diagnose diseases, disorders, injuries and other related health conditions (WHO, 2017). Although this system is helpful, it is problematic from a global health perspective because this system gives priority to the biomedical model, but is devoid of the contexts in which the individual lives; familial and community relationships; environmental connectedness; and, religious and cultural beliefs and traditions. The biomedical model fits well in a neoliberal culture, but is in conflict with a Cosmo-centric, anthropocentric, and community-centred cultures where illness is defined by additional factors (Wolffers, 2000). Trying to push for an autonomous decision by an individual would be counterproductive, therefore an approach where human dignity is valued is highly recommended. The principle of respect for human dignity holds a prominent position in the intergovernmental instruments dealing with bioethics that have been adopted during the last decade. The emphasis on human dignity is known as “the shaping principle” of international bioethics (Andorno, 2009). The value of the aforementioned for ethicists lies in the fact that any discussion about treatment options/plans/values needs to include reference to all the care providers and necessary community members (including traditional healers, elders, etc.) in order to build trust in the treatment plan suggested by the secular physician/healer, when having conversations about important healthcare decisions. As mentioned before, for members of the indigenous groups studied, no illness is one dimensional (including only the bodily aspects), but additionally includes spheres of the spiritual, emotional, relational, and environmental. Because of this healing is seen as multi-faceted and should therefore address the various integrated levels important to indigenous cultures. Healing needs to take place on various levels in order to restore a state of equilibrium and balance between the different spheres. Since members of the indigenous groups studied see themselves as a part of a larger entity, healing often needs to be guided by a special traditional healer. The traditional healer mainly uses elements from the immediate environment and also addresses the illness of the individual on different levels. Having said this, the results also indicate that none of the indigenous groups would oppose it if the individual sought complementary assistance from the western healthcare system if the illness was seen to be one that would respond to western medicine. The two systems are often seen as complementary to each other as the groups believe both play important roles by addressing different aspects of the healing process.

Subsequently the traditional healer is connected to the ancestral spirits and/or OTHPs and it is not customary for members of indigenous groups to doubt the remedies/actions proposed by the healer. Since it is not only the healer prescribing the action, but the spirits, the individual will not oppose the care plan. Consequently, this perspective challenges the philosophical (and legal) tenets of autonomous decision-making for healthcare choices. The same approach/mind-set applies when an individual, from one of the indigenous groups, seeks complimentary western health care. The individual will present his/her symptoms and may adhere to the prescribed treatment without question, provided it is acceptable to the traditional healer. Since the traditional healer is the one in a trust position with the group and the individual, the task of primary healer/caregiver will be bestowed on them. The individual will not oppose the treatment plan set forward by the western practitioner, but may take it to the traditional healer to confirm that the course of action would not upset any equilibrium. It is important for western practitioners to take cognizance of this as it may influence the individual’s ability to execute the care plan; commit to therapies; or even see it as an important aspect of their healing.

## 5. Conclusion

The problem faced when addressing health disparities and ethical dilemmas in a global context, from only a western view, is that there are many excluded circumstances (e.g. culture, religion, economics, etc.) that need different approaches and learned understanding (Hellsten, 2008), even within a dominant culture. This study highlights that health decision-making is good (in the sense of Plato’s form or idea) if it is strengthened by individuals’ ability to fulfil their moral and ethical responsibilities towards the group and a supreme being. Therefore, the notion of autonomy, as presented as a critical value in western medicine, has a very different definition in the indigenous cultures studied. Autonomy must be seen as the individual’s ability to make a choice that serves his family/clan/community.

As healthcare at large has been confronted with demands for diversity and inclusivity, it is our thesis that global health could benefit greatly from global bioethics as an intersection between differing value-systems to create justice and beneficence and to achieve the highest attainable standard of health as set forward by the United Nation’s Sustainable Development Goals (2015). Indigenous perspectives on the values of interrelationships between different aspects of a person and their environment, as well as the importance of belonging to a group, could challenge the notions of a dominant western perspective. We must consider that individual choices can have an impact beyond just the individual. Therefore from a social justice perspective, ethical healthcare includes efforts to learn about and gain a better understanding about what people from various indigenous cultural backgrounds see as important when making decisions. Often understanding indigenous cultural values requires a lifetime of effort and attention. The indigenous experts who reviewed the results hold that the aforementioned is in line with their cultural practices, but also ascertain that it is imperative to mention that urbanization has influenced health care behaviour in many members who have moved to cities, and that the aforementioned discussion and conclusions pertains in specific to those who still live in rural and tribal areas. To this point then, when a member of an indigenous group seeks healthcare in an urban area (or away from their home community) the applicability of this thesis could be limited due to time and resource constraints within the healthcare system, especially during a pandemic. Furthermore, policy and practices changes such as visitation restrictions during COVID-19 hospitalizations can further limit opportunities for indigenous patients to be ideally supported in culturally relevant ways. However, efforts should be made to connect the patient and their community members in order to assist the patient with making the best health care decisions possible.

The biggest contribution this research can make to the knowledge field of global bioethics is that decision-making is a complex journey. There are many commonalities among the three indigenous cultures discussed here; in particular, that decision-making should not be considered as a linear process with a conversation/dialogue between just two individuals. Rather it is a process which involves a whole, a cosmological interconnected whole, that occurs between those who have come before and those who will follow, and must acknowledge the role one plays in society at the present. Decisions must be considered from a multi-layered perspective and are influenced by three time spheres.

Finally, it is important to acknowledge that there are indeed many differences between the values and perspectives of the three cultural groups studied in this meta-narrative. It is by no implication of this discussion to suggest that the three cultures are homogenous in all aspects of their views of the Cosmos, personhood, and/or healthcare decisions. Neither should it be construed that all indigenous cultures are equated to similarity in their views on healthcare choices and decision-making. This study was merely an attempt to enquire about whether there were indeed similarities between the three cultures studied and to explore how this may better inform the ongoing discourse in global bioethics.
